# Atteinte œsophagienne au cours du pemphigus vulgaire

**DOI:** 10.11604/pamj.2014.17.118.2452

**Published:** 2014-02-18

**Authors:** Wafae Raffas, Badreddine Hassam

**Affiliations:** 1 Service de Dermatologie, CHU Ibn Sina, Université Med V, Souissi, Rabat, Maroc

**Keywords:** Oesophage, pemphigus vulgaire, muqueuse digestive, esophagus, pemphigus vulgaris, gastrointestinal mucosa

## Image en medicine

Le pemphigus vulgaire est une maladie bulleuse auto-immune rare de la peau et des muqueuses. L'atteinte oesophagienne y est le plus souvent méconnue bien que sa prévalence soit estimée à 70%. Elle peut être asymptomatique de découverte endoscopique, ou encore isolée constituant la seule manifestation du pemphigus. L'examen endoscopique est dénué de risque et permet par la biopsie de faire la distinction entre une atteinte oesophagienne du pemphigus et une oesophagite candidosique ou herpétique chez des patients sous traitement prolongé par immunosuppresseurs. Par ailleurs, la sévérité des lésions oesophagiennes peut justifier de fortes doses de corticoïdes alors même que les lésions cutanées sont minimes. Une fibroscopie de contrôle est préconisée après traitement à la recherche de complications telle qu'une sténose oesophagienne. Nous rapportons le cas de Mme S.A., 32 ans, suivie pour un pemphigus vulgaire depuis 5 ans, traitée par corticothérapie orale et azathioprine. Elle était hospitalisée pour rechute au niveau de la muqueuse buccale survenue lors de la dégression de la corticothérapie. Elle rapportait par ailleurs des épigastralgies, avec des douleurs rétrosternales partiellement soulagées par un traitement anti-sécrétoire. Une fibroscopie oesogastro-duodénale était réalisée objectivant un aspect d'oesophagite mycosique, une gastrite érythémato-pétéchiale et une bulbite congestive; l’étude anatomopathologique des biopsies étagées révélait un clivage intra-épithélial de la muqueuse oesophagienne sans signe d'atteinte mycosique. Une augmentation de la corticothérapie à 2mg/kg/j associée à l'azathioprine, et à un traitement protecteur de la muqueuse digestive anti-sécrétoire et antiacide, ont permis un amendement de la symptomatologie digestive.

**Figure 1:**
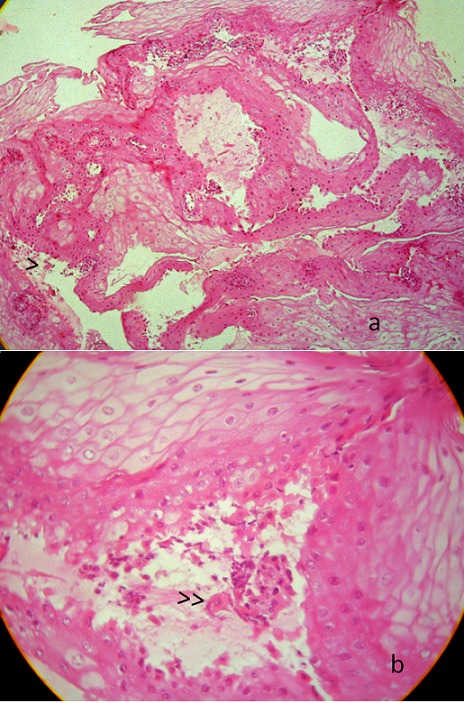
(a,b): clivage intra-épithélial (>) et cellules acantholytiques (> > ) à l'examen histologique de la muqueuse œsophagienne

